# “To Antipolis, my sisters!”: ETSI as a forum of contestation, collaboration and orchestration

**DOI:** 10.1080/13511610.2024.2379806

**Published:** 2024-08-17

**Authors:** Panagiotis Delimatsis, Zuno Verghese

**Affiliations:** Tilburg Law and Economics Center (TILEC) and Tilburg Institute for Law, Technology and Society (TILT), Tilburg Law School, Tilburg University, Tilburg, The Netherlands

**Keywords:** ETSI, resilience, legitimacy, crisis, contestation, orchestration, standardization‌

## Abstract

Over the years, ETSI, the prime European standard-setter for ICT standards, has experienced significant critique about its legitimacy, including more recently by the European Commission in the 2022 Standardization Strategy, which focused on the alleged deficiencies of its global participation model. Despite potential missteps in certain instances, ETSI has overall addressed such critique with remarkable success. In this article, we argue that this occurred thanks to various strategies that ETSI used to sustain its organizational resilience over the years. In regard to its interaction with its de facto controller, the European Commission, we argue that ETSI had recourse to three key resilience strategies: one where ETSI took a hard stance resisting to the Commission's desires (contestation), one where ETSI intentionally succumbed to the Commission's desires or preferences (orchestration)**,** and one where ETSI opted for a reconciliatory approach to achieve commonly set objectives serving the mutual interest (collaboration). Interestingly, such strategies were not only resilience-enhancing, but also legitimacy-conducive. This article contributes to two different strings of academic literature relating to organizational resilience, on the one hand, and institutional legitimacy, on the other, by combining them in a manner that, to the best of our knowledge, had not been done before in the relevant literature. In the discussion section, we draw observations that are pertinent to discussions and developments since the 2022 European Union’s Standardization Strategy.

## 1. Introduction

The economic, social and environmental importance of technical standards has continued growing apace over the last three decades. This was mainly driven by globalization, technological advances in the industry of information and communication technologies (ICT) and the internationalization of supply chains. However, institutional advances at the multilateral level (notably the adoption of the Technical Barriers to Trade Agreement-TBT – at the World Trade Organization-WTO) have further boosted the importance of participation and international collaboration in promulgating international standards within standard-setting organizations (SSOs) who are open to stakeholders regardless of their seat or place of activity. Technical excellence has grown to matter more than any other criterion regarding participation in and contribution to the work of such SSOs. A functional approach of this type also came to challenge excessive institutional formalism: consortia and informal SSOs have often become much more important – and arguably effective – generators of technical standards than formal national and international standards development organizations (Biddle 2017; Delimatsis, Kanevskaia, and Verghese 2021).

Be it formal or informal, SSOs have in essence three crucial functions: first, they allow for learning about and certifying the value of (new or improved) functionalities or combinations thereof (the discovery function). Second, they direct market expectations towards a given technology, for instance, through promoting adoption and diffusion (the standardization function). Third, they regulate and govern the relevant market by requiring the owners of patents essential to the standard (so-called “standard-essential patents” or SEPs) to grant use licenses on fair, reasonable and non-discriminatory (FRAND) terms (Gandal and Régibeau 2015; Lerner and Tirole 2015). In that respect, SSOs act as important coordination devices that can lead, at least in theory, to substantial efficient outcomes (Farrell and Saloner 1988). SSOs can potentially resolve conflicts, for instance, through repeated interaction of competing vested interests (Larouche and Schuett 2019). At the same time, as important arbiters of the technical superiority of a given technology, SSOs can determine winners and losers in technological battles (Delimatsis 2018; Kanevskaia 2023; Rysman and Simcoe 2008). The important financial resources that are invested by companies to participate actively in SSOs but also the increase of SEPs-related patent litigation and antitrust enforcement are all indicative of SSO prominence (Delimatsis 2019). These observations corroborate the view that neutrality, good governance and the application of relevant legal safeguards is crucial for SSOs.

While most of the standards produced by SSOs are of a voluntary nature, such standards can often become mandatory through their use in State regulations by reference (Schepel 2005; Sheffner 2019). In that case, standards are incorporated in a public legislative instrument and through this incorporation acquire regulatory standing and a full legally binding value with important market effects (*Stichting Rookpreventie Jeugd and Others v Staatssecretaris van Volksgezondheid, Welzijn en Sport* 2022). In that sense, the stakes relating to the work of SSOs are not only significant from an economic but also a political and legal perspective (Delimatsis 2015), raising questions of legitimacy and accountability internally at the organizational level of an SSO and externally, vis-à-vis standards users, including consumers, businesses and governmental agencies (Freeman 2000; Van Gestel and Micklitz 2013).

The European Union (EU)’s New Approach has been the EU’s hallmark legislative technique to overcome technical barriers to trade within the EU but also improve the EU’s competitiveness as a whole (European Commission 1985; European Council 1985). Under this hybrid regulatory technique, Community legislation limits itself to formulating essential health and safety requirements and is complemented by the promulgation of harmonized European standards that specify the technical characteristics of products adopted by the European standard-setting organizations (ESOs) upon a mandate by the European Commission. The European Committee for Standardization (CEN), the European Committee for Electrotechnical Standardization (CENELEC) and the European Telecommunications Standards Institute (ETSI) would be instrumentalized to co-shape legislation for the smooth functioning of the EU internal market: although standards were still considered as private and voluntary, their inclusion in public regulatory instruments as benchmarks that ensure a presumption of conformity with those instruments would resolutely change their legal nature (Schepel 2015). Such delegation of regulatory tasks could be viewed as problematic at best – and potentially contradicting EU law at worst (Falke 1997).

The contribution of ESOs to the success of the internal market for goods (and services) has been crucial, with over 20% of European standards being harmonized standards in 2011 (European Commission 2011). For our purposes, ETSI in particular has been an offspring of the single market project in mid-90s but also a key driver of this EU project in the area of ICT (David and Shurmer 1996). ETSI’s contribution to lowering technical barriers to trade within the EU, thereby reducing compliance costs, improving network effects and interoperability issues but also ensuring a global competitive edge for European standards through its open membership model and its cooperation with international SSOs is well-documented (Bekkers 2001; Cantero Gamito 2018). ETSI has also been the key implementer of the EU’s priorities regarding ICT standardization, from 5G, to cloud computing to IoT and cybersecurity, either alone or by joining forces with the other ESOs and additional issue-specific agencies.

At least for two decades and driven by the ever-increasing importance of the EU as a big consumer market and significant global ICT market regulator, ETSI has been the intellectual hub for technical standardization, a sort of promised land where engineers, lobbyists, National Standards Organizations (NSOs) and market participants alike would meet, thereby giving the EU a unique opportunity to get a leading position in shaping the preferred technologies of tomorrow. ETSI’s open method of standardization, which, unlike within CEN and CENELEC, allows companies to participate directly in the development of standards, was arguably more aligned with basic principles of international standardization, including transparency, openness, effectiveness and relevance (the so-called “TBT principles”) (WTO Committee on Technical Barriers to Trade (TBT Committee) 2000). However, as the EU has moved towards spelling out its ambitions for technological sovereignty, its willingness to reduce dependencies and to protect its democratic values in recent years (European Commission 2020), the EU standardization system is set to undergo an important transformation. In this framework, ETSI is at the spotlight of the new EU standardization policy due to its open membership system, whereby (inter alia, Chinese or American) firms participate directly in standards development (European Commission 2022a, 1). There appears to be a gradual disenchantment with ETSI’s decision-making process.

This is an interesting policy development as it shakes further the European standardization edifice, culminating a decade of challenging events for SSOs: first, two judgments by the Court of Justice of the European Union, *Fra.bo SpA v Deutsche Vereinigung des Gas- und Wasserfaches eV (DVGW) — Technisch-Wissenschaftlicher Verein* (2012) and *James Elliott Construction Limited v Irish Asphalt Limited* (2016), led the attack to the voluntary (and private law) character of standards, suggesting that national and European (harmonized) standards can produce regulatory effects akin to State legislation, notably when incorporated into national or European legislation. Through this incorporation, these standards become part of the legislation they belong to (State law or EU law, respectively). Between the two judgments, the adoption of the EU Standardization Regulation (EU Regulation 2012) opened the door for challenging harmonized European standards once they were published in the EU Official Journal, as it upgraded their legal status by clarifying the allegedly substantial monitoring role of the European Commission but also gave the possibility to Member States and the European Parliament to challenge harmonized standards (Schepel 2013). These events challenged the *institutional* legitimacy of SSOs as *voluntary* private rule-makers and put the spotlight on their procedural integrity, including their inclusiveness, transparency and due process.

The more recent call made by the European Commission to ETSI to amend its open membership rules when promulgating harmonized standards constitutes a challenge to ETSI’s *substantive* legitimacy. The EU standardization strategy (European Commission 2022a, 4) was adamant about the strategic importance of certain standards for the EU’s interests and the ensuing possibility of foreign interference into the EU machinery of producing harmonized standards by corporate interests which do not share the EU’s democratic values. As a result, ETSI had to comply with a regulatory amendment to Regulation 1025/2012 (European Regulation 2022) by changing its governance rules to limit participation in the creation of harmonized European standards to NSOs (ETSI 2023).

Against this backdrop, the objective of this article is to offer an analytical framework for assessing the interaction between the European Commission and ETSI, a key developer of EU harmonized standards in the public interest, in a time period of over thirty years. More specifically, we establish a crude taxonomy of key resilience strategies that ETSI has applied over the years to confront its immediate institutional public controller, the European Commission. Based on previous empirical research (notably semi-structured interviews with experts associated with ETSI in various time periods), we assume that these challenging moments of interaction questioned ETSI’s organizational resilience and legitimacy. Through our research, we have identified three main strategies that ETSI applied to overcome such moments: the first relates to contestation, whereby ETSI took a hard stance towards the EU’s demands; the second relates to orchestration, whereby the Commission, acting as the orchestrator in this case enlisted and steered ETSI which, acting as the required intermediary, promulgated standards to pursue common policy goals. Finally, the third strategy identified relates to collaboration, whereby ETSI and the Commission collaborated as two economic actors of equal importance to advance a common objective through standard-setting.

We submit that assessing ETSI’s legitimacy is a dynamic endeavour that can be conducted based on this analytical framework we introduce. Its decision to contest public authority; its acceptance to be enlisted; or its willingness to collaborate demonstrate, in our view, key traits of ETSI’s governance over time, including agile and responsive management, effective internal communication, active leadership, responsible administration and institutional mutability (Delimatsis 2023a). However, assessing such causality goes beyond the scope of this article (for an effort in that direction, see Stanojević 2024). Still, our objective is to steer further empirical research over such instances that made ETSI a more resilient private organization. We make the first step by shedding light on three examples of such strategies and how they relate to ETSI’s lasting legitimacy. Whilst there are historical accounts of these critical junctures we identify, existing studies do not account for how ETSI, while in dialogue with the EU Commission, by responding and adapting to changes and (legitimacy) challenges, both internal and external, sustained its organizational resilience. By taking this approach, this article sheds light on the implications of the resilience strategies used by ETSI for its legitimacy (and authority) in the ever-evolving field of standardization but also offers broader insights relating to the interaction between public authorities and private regulatory institutions.

## 2. Conceptual framework

In what follows, we will discuss the conceptual framework that we apply to examine how ETSI as a developer of “harmonized” standards in the public interest, employed the three previously identified tactics (contestation, orchestration, and collaboration) in its interaction with the European Commission during critical moments in its history.

The modalities of interaction under which these strategies are employed are outlined in more detail below, drawing on literature from economic regulation and governance. These strategies are associated with certain organizational changes within ETSI, and its resilience. For the analysis of these evolutionary dynamics, resilience and legitimacy of ETSI, we rely on the conceptual framework provided below.

### 2.1 ETSI’s interactions with the EU

Since the EU New Approach, the EU Commission cooperates with ETSI as part of what is referred to as a co-regulatory regime (Alexandridis 2019, 14; Cantero Gamito 2018). When it comes to the promulgation of harmonized standards, the Commission acts as principal (in that it designs the overarching goals of a given technical standard, typically as part of a Directive or Regulation), and ETSI as the agent (in that it defines and specifies the technical content of a given standard to be used for regulatory purposes). The influence that the public regulator can exert over ETSI’s substance and procedures, at least where the development of these “harmonized” standards in the public interest are concerned, puts some restraint on its regulatory autonomy. However, ESOs can reject such standardization mandates. If accepted, then there needs to be a genuine relationship between the mandate and the developed harmonized standard. For instance, the CJEU clarified in *Anstar* that the mandate is important to consider when interpreting the scope of a harmonized standard (*Anstar Oy vs Turvallisuus-ja kemikaalivirasto (Tukes)* 2017). Once the mandate is accepted, the Commission cannot interfere with an ESO’s internal decision-making procedures nor with the content of the standards they create (European Commission 2015, 14–15). This, however, does not constitute a *carte blanche* for the ESOs, as they have important obligations, including acting in the public interest when developing standards (European Commission 2003, 10–11).

Since 2012, and the enactment of Regulation 1025/2012, a legislative framework was put in place that governs ETSI’s relations of cooperation with the EU Commission, which introduced *inter alia* certain requirements for the standards development process for harmonized standards and a control mechanism to enhance the public oversight of the ESO’s activities (Eliantonio and Volpato 2022, 6). The ECJ’s decision in the James Elliott Construction Limited v Irish Asphalt Limited (2016) case, which held that harmonized standards are part of EU law, made the public nature of harmonized standardization apparent and resulted in further restraints, namely the ESOs having to respect essential constitutional requirements and general principles enshrined in the Treaties (Senden 2017).

Since *James Elliott Construction Limited v Irish Asphalt Limited* (2016), a broader discussion about the repercussions of the juridification of EU harmonized standards and the constitutionalization of the EU standardization system has emerged. Such discussion also created a certain tension between the Commission and ESOs, notably ETSI. Initially, the discussion focused on the impact of judicial review on the private nature of standardization. More recently, the focus shifted on who participates in standard-setting within the EU. In this respect, one of the most important factors for the success of ETSI was also considered by the EU bureaucracy as one of its most crucial weaknesses: its open membership and ensuing weighted voting policy, which appeared to give undue influence to third-country participants but also diminish the role of SMEs, civil society and users (European Commission 2022a, 4).

This increased intervention within ESOs internal affairs on the side of the Commission coincided with a time of increased geopolitical competition, techno-nationalism, strategic considerations entering regulatory policy and fierce competition by private standard-setters. While the European Commission underlined the importance of promoting the EU values and the public interest in ETSI’s standardization activities, others have warned that changes in the ETSI functioning and rules of procedure may disincentivize market participants, including from third countries, to participate in the development of all standards within ETSI and instead lead them to flee towards non-ESOs, thereby reducing the EU’s role and influence in the international ICT standards ecosystem (European Commission 2022b). In addition, a report on the ESOs’ compliance with Regulation 1025/2012 found that undue influence by third-country companies within ETSI may not have been as significant as suggested (European Commission 2021).

### 2.2 Models of interaction

The models of interaction between the EU and private regulators can take different forms.^1^ The present analysis relies on the following three models of interaction to theorize about ETSI’s institutional legitimacy and resilience: contestation, orchestration and collaboration. We assume that the relationship between the European Commission and ESOs has evolved from one of delegation, whereby the EU would only use the implementing capacity of ESOs, to a more entangled government partnership, which has made this relationship – and by implication, the interaction between the public and the private actor – more sophisticated. ETSI, for instance, is not only part of the implementation of rules, but also has an active role in the content and shape of technical standards, as well as their monitoring and enforcement. In what follows, we describe what we consider as the constituent elements of these three models.

**Contestation** can be understood as covering a range of practices, which express disapproval or disagreement against certain norms, practices, demands or expectations in a direct or indirect manner (Wiener 2014). Contestation in global governance relates to challenges against allegations of power and hierarchy (Zürn 2018). Different forms of contestation in transnational private regulation are also well documented (Bartley 2021). The relationship of ESOs with the European institutions and notably the EU executive reveals a pattern of authority relationship that produces conflict, contestation and often resistance, as we show below.

This is notably because the two sides are embedded in a key normative order for the EU, that is, the EU standardization ecosystem, both producing key inputs and outputs: the Commission produces mandates as a key input and publishes standards as a key output. On the other hand, ESOs put in place the infrastructure for producing standards but then also are the key actors for the promulgation of standards. As ESOs also have to please their constituents, their interaction with the Commission can produce contestation against practices, existing or proposed arrangements, and demands originating in the EU executive. Viewed as an expression of power over the ESOs, such practices can be fiercely contested and fuel conflict, creating critical junctures that may even lead to resistance against cooperation and thus gridlocks in the creation of standards. These in turn can impede the EU legislative process; without standards, the EU legislative instruments that call for their creation cannot be fully implemented and enforced, creating market uncertainty.

ESO contestation is also to be expected as exiting the ESO is not really an option for its constituents due to its monopoly nature as a provider of (harmonized) European standards, which makes voice as the only credible option for ESO constituents. As a final point, we submit that contestation is directly linked to the likelihood of non-incremental institutional change, which in some cases can lead to the decline and emergence of new institutions. However, in the case of ESOs, internal change of processes, rules and practices – rather than exit – appears to be the only solution due to their idiosyncratic role in the EU economic policymaking.

**Orchestration** typically entails the mobilization of an intermediary by a principal or orchestrator (the State or its agents) who is enrolled to pursue a common policy goal (Abbott et al. 2016). Orchestration has been studied in various sectors and issue-areas but the common denominator is the identification of a public actor who seeks support for solving a problem of public nature by enrolling a private economic actor. In that sense, orchestration suggests that, in reality, the public and private cannot be conventionally distinct but intermingle.

In this process, the orchestrator initiates, guides, broadens and strengthens regulatory activities by certain private actors. As the entrepreneurial public actor reaches its objective through an intermediary, orchestration is a hybrid, indirect form of governance in a given sector or issue-area that addresses collective action problems. Orchestration is also typically a soft form of governance, as the public actor at issue cannot exercise coercion or direct control on the enrolled intermediary; rather, it will have to employ ideational or material incentives to mobilize and instigate the cooperation of the intermediary (Abbott et al. 2015). The use of both facilitative and directive instruments (notably a credible regulatory threat) can determine the success of orchestration (Henriksen and Ponte 2018).

In this respect, orchestration is both bottom-up and top-down, and must ultimately respond to the demands of all parties involved (Hale and Roger 2014). Such a partnership constitutes a distinct modality of public action, allowing public authorities such as the European Commission to expand their regulatory capacity in areas whereby the technical knowledge required is located elsewhere. Orchestration may have significant distributional implications in cases whereby there is variety in the group of private partners; a private actor is legitimized by being enlisted by the public actor, while those that are not chosen may suffer a de-legitimization shock.

Finally, **collaboration** is a desirable method whereby an active, voluntary and conscious partnership between a public and private actor occurs. Collaboration presupposes a (virtually) horizontal power relationship, suggesting that the public and private actors involved recognize each other’s authority and valuable contribution to the pursuit of the commonly established objective (Cashore et al. 2021). Thus, communicating directly and working together in a synergistic fashion reinforces the likelihood of successfully attaining the goal set out by the partners. Collaboration as a mode of interaction is a balanced governance partnership that is based on functionalist premises (Mattli and Seddon 2015).

To a certain extent, collaboration is a key form of co-governance, which when institutionalized, can lead to clearly delineated competences, mutual respect and a certain level of congruence between the functioning of private and public actors at issue (Tosun, Koos, and Shore 2016). Collaboration, as used in our analytical framework, is close to the concept of “jazz”, which dismisses orchestration as a hierarchical public-private relationship and instead advances the idea that private and public actors are often just accommodating to one another, which means that in some cases the one or the other will respond or lead, depending on the case and circumstances at issue (Knudsen and Moon 2017).

### 2.3 A synthesis of interaction models

Having presented the three models of interaction, it is important to note that these models of interaction are not cast in stone; rather, depending on the circumstances, the specific regulatory arrangements, the alignment of goals of the different actor groups, but also the strategic self-interest at any given point in time, the interaction among public and private actors will change from one model to the other; in theory, it is even conceivable that until the final goal is achieved, the interaction will involve more than one interaction models (an initial phase of contestation will be succeeded by a phase of collaboration). What is certain is that none of the drawbacks of each model of interaction will prevent co-working. This is because the main premise for such co-working is the high transaction costs but also the advantages that each partner has: the technical knowledge in the case of private actor and the veil of legitimacy and power of coercion on the side of the public actor. Thus, regulatory outsourcing cannot be avoided, even if its modalities will vary over time, space and issue-area.

Other than showing that it is reductive to conceptualize the interaction of public action and private authority as complementary or competitive, all three modes of interaction underscore their ubiquitous relationship with legitimacy and legitimation. For now, one should keep in mind that ESOs should not just feed their external legitimacy; internal legitimacy, i.e. the acceptance by its membership of ETSI’s right to set standards is equally (if not more) important. All three modes of interaction between ETSI and the European Commission, for instance, can also be viewed as legitimation strategies: contestation is a concerted reaction that often builds in a bottom-up manner and, when expressed, offers satisfaction to ETSI’s membership; orchestration legitimizes ETSI by making it the preferred private partner to steer among a variety of other possible private partners; finally, collaboration results in recognition by a public actor of the important role that ETSI can play in achieving public goals, which means that there is a recognition of its legitimacy as a standard-setter in the issue-area at stake.

Finally, an important point that ensues from the study on the interaction of ETSI with the Commission is that, just as the European Commission will seek to shape private authority, ETSI will also seek to shape (and increase its leverage in shaping) public action through its standard-setting activity, both in terms of priority-setting and regulatory choices. This may even lead to ETSI proactively seeking to be active in certain regulatory areas, which can result in ETSI expanding its mission (Delimatsis 2023a). Viewed from this lens, ETSI and the European Commission constitute a composite site of political authority whereby a certain fluidity of authority contributes to the resilience of the EU technical standardization ecosystem.

### 2.4 Critical moments, resilience strategies and legitimacy

In our analytical framework, the existence of inflection points or critical moments is important in allowing the three strategies discussed above to emerge. In our view, such instances encompass extraordinary moments and erratic events (or sequence thereof) which are potential threats in the status quo or opportunities to improve the position of the private actor (for example, by allowing it to expand its regulatory activities). Crises of this type can lead to non-incremental institutional change, opening up a new window of opportunity for the private organization (Delimatsis 2023b). They are testbeds for effective management and active leadership. Crises can be one-off instances or grow gradually (for example, out of ever-increasing dissatisfaction with the dealings of the management team that can entail a growing sense of de-legitimisation). Crises of this type require critical decision-making under conditions of uncertainty and time pressure. The link between crises or disturbances, crisis management and resilience is then quite straightforward: just as recovery presupposes a crisis, resilience builds on a disturbance that instigates a moment of stress, which can be overcome after the system displays its panoply of resilience-causing traits. Thus, resilience and crises are two concepts in repeated interaction that share similar characteristics (Delimatsis, Bijlmakers, and Borowicz 2023).

ETSI is a member-driven organization. ETSI’s membership comprises of a heterogenous group of actors and interests. Internal pressures can slow down and impede effective decision-making, while external pressures can challenge ETSI’s position as the prime standard-setter of ICT-related standards. Addressing one type of pressure to increase legitimacy may often be in direct opposition to the reaction needed to address the other type of pressure, which also affects the organization’s legitimacy (Black 2008, 157–158). This can cause difficulties especially during crisis moments when decisions must be taken rapidly. To the extent that ETSI faces conflicting legitimacy demands, and rising tensions require it to make trade-offs that favour the interest of one community over another, ETSI may be said to encounter a “legitimacy dilemma” (157–158). These internal dynamics and tensions can result in an organization experiencing a “legitimacy crisis”. If this crisis is managed, it may lead to resilience, if not, it can lead to disaster.

ETSI’s enactment of crisis management strategies during crisis moments may have implications for the organization’s legitimacy. The outcome can be legitimacy-enhancing where the changes positively affect the organization’s normative/political legitimacy, or because they respond to and meet certain legitimacy demands, affecting stakeholder’s perceptions and evaluations of ETSI. The crisis management strategies enacted by ETSI during crisis moments and their implications may affect the EU’s perception of ETSI’s legitimacy and trustworthiness (Roux-Dufort and Lalonde 2013). The EU’s acceptance of ETSI’s authority to develop European standards might be reinforced or legitimized after a crisis episode. ETSI’s enactment of crisis management strategies during crisis moments may have implications for its embedment, legitimacy and relative influence in an ecology of multiple private standard-setting organizations.

## 3 ETSI’s resilience strategies

### 3.1 Methodology

The empirical data for this article are part of a broader research project, which studies the enabling features, traits and strategies that allow private regulatory bodies in Europe to endure and retain relevance despite multiple exogenous challenges.^2^ We opted for case study research, as it allows to investigate in detail how different private and hybrid regulatory institutions use standards as a key mechanism to display resilience over time. In this particular instance relating to ETSI, the case study research method allowed for investigating how the external challenges and internal pressure are perceived by ETSI and how the institution navigates through these challenges to enhance its resilience and legitimacy over time.

For this paper, the second author conducted fieldwork at the ETSI premises. He also conducted and analysed transcripts of audio recordings of 10 semi-structured interviews with key actors at the highest level and with extensive ETSI involvement in the last twenty years. The temporal siting of the case studies, and the topicality required that the interviewees hail from diverse backgrounds in terms of their contributions within ETSI and duration of involvement, and area of specialization (for example, in technical aspects or in governance). For the empirical part of this article, we further relied on the perusal and analysis of ETSI documentation. In particular, we relied on publicly available documents for the SIM Lock Case, in overcoming the limitations of inadequacy of interview data (including off-the-record ones).

In our empirical part, we identify triggering events that could potentially lead (or have led) to a type of critical moment and discuss how the three strategies we identify were used to enhance ETSI’s resilience. As the following case studies will demonstrate, the strategies enacted by ETSI during crisis moments affect how the EU regulator perceives ETSI and behaves in its regulatory activity. In that sense, the discussed strategies also offer a window to look into ETSI decision-making under conditions of uncertainty and time pressure (Wolbers, Kuipers, and Boin 2021, 375).

The case studies also shed light on how ETSI’s strategies are legitimized and contested in ways that shape perceptions of ETSI, by the EU and other stakeholders. ETSI might act proactively to consolidate and expand its competences. Our work also identifies a paradox that speaks to the fragility of private governance: the more the legitimacy of a private organization such as ETSI increases, the more prone to and fragile against internal and external contestation that organization can be. To address this fate of private governance, private organizations have to be alert and responsive, always catering for adequate strategies to anticipate conflicts and improve their resilience.

The three strategies ETSI employed constitute a concerted attempt to influence the outcome of these episodes that triggered their use. The first strategy emphasizes the importance of ETSI’s unique governance model in ensuring the availability of ETSI standards and their global relevance in the ICT standardization field (ETSI Membership Model). The strategy of contestation discussed here evolves around ETSI’s hard stance resisting to the Commission’s desires. The second strategy focuses on ETSI’s alignment of its policy with the EU’s preferences upon its request and legal compliance therewith (ETSI’s IPR Policy). The strategy of orchestration discussed here deals with ETSI’s intentional enrolment to the Commission’s goals (orchestration). Finally, the third strategy spotlights ETSI’s responding to and working with the EU Commission to find a solution to a problem of mutual interest (The GSM “SIM Lock” Case). This strategy of collaboration that we examine underscores ETSI’s reconciliatory approach to achieve commonly set objectives serving the mutual interest.

### 3.2 Case study for contestation – membership model

Since its inception in 1988, ETSI has been operationally and institutionally distinct from its peer ESOs in several aspects, but most importantly due to its direct participation model, and in relation to this, the relevance accrued to NSOs within ETSI. This distinctness of ETSI has often led to ETSI having to explain and justify its unique set-up to the European Commission and other stakeholders, typically when the European Commission is in the pursuit of reforming the European standardization system.

We identify two such moments – one during ETSI’s inception and the other in the 2000s. During these moments, we note that ETSI finds itself under pressure to respond and adapt to the European Commission’s evolving vision driven by the goal of realizing a cohesive standardization system that serves the Community as well as public interest goals. During these episodes, ETSI is compared to CEN and CENELEC on specific institutional parameters and performance, with the degree of involvement of the NSOs within ETSI emerging as an important point of contention for the European Commission. The responses by ETSI show that it has consistently pursued a strategy of contestation in defending itself against the influence and demands of the European Commission. The case study sheds light on how ETSI’s decision to preserve its participation model is legitimated and contested in ways that shape perceptions of ETSI and its legitimacy by the EU and other stakeholders.

#### 3.2.1 Through inception and formative years: 1987 until 1994

In its 1990 Green Paper, the European Commission acknowledged that ETSI’s creation “represented a radical change in approach to the European Standardisation System”, specifically noting ETSI’s direct participation by “all interested parties in standardization work rather than for representation through national delegations headed by the national standards body” as was the case within CEN and CENELEC (European Commission 1990, 16). ETSI’s opting for this model was driven by its worldview that each member be recognized as an individual economic actor for whom the ability to directly participate and represent their own interests in the making of voluntary formal standards would be both effective and efficient (Temple 1991, 52 and 84). This direct participation model caused much unease amongst the NSOs since it implied that technical contributions would bypass “the national standards production machinery”, thus lowering the involvement and relevance of the NSOs (52).

ETSI’s inception was accomplished within ten months of the European Commission’s proposal in June 1987 – this swiftness was laced with a lack of procedural clarity in key operational and decision-making aspects of ETSI’s management of standards development activities in its early years (see for example the section on IPR Policy). Temple notes that among the most problematic items for discussion during ETSI’s first GA meeting in 1988 related to the approval of standards, specifically the rules for work item initiation and approval of standards, and the degree of coverage of national voting for decisions. During this first GA meeting, ETSI decided on tasking a “small group” to consider the appropriate revisions for the rules on the standards approval process (53–54, and 10–11). Temple also notes that the lack of clarity on this set of rules was intimately related to the role and relevance of NSOs in the ETSI’s envisioned process for standards development (52).

Finally, at the time of its creation, ETSI as a new body did not enjoy formal recognition as an ESO within the then European standardisation system yet. ETSI’s recognition as an ESO would take effect only after 1992. An important cause for the delay was the sustained dissatisfaction of the European Commission with ETSI’s rules. Importantly, the European Commission was of the opinion that ETSI’s rules did not meet the basic principles of standardization, namely “transparency, openness, consensus, independence of vested interests, efficiency and decision-taking on the basis of national representations”, which the European Commission deemed were crucial for European standardisation to serve public interest goals (European Commission, Schepel, and Falke 2000, 16).

Regarding ETSI’s perspectives on membership, an important consideration was that historically, NSOs had a negligible role to play in the development of standards in the field of telecommunications. Moreover, ETSI claimed that the direct participation model enabled a resource-efficient “one-stage process” for its European members. ETSI’s European members could mobilize and submit their contributions directly at the European level. These members were thus no longer solely dependent on investing resources within the confines of the national delegation model. Furthermore, ETSI’s “logic”/ “justification” of perceiving its members as economic actors was also supplemented by its internal considerations of assigning the highest value to expert contributions from industry and it being reflective of values that are consistent with the contexts of its creation – an arena where industry actors could participate as equals with governmental and state agencies.

The European Commission was insistent upon ensuring that the NSOs enjoyed visibility and legitimacy within ETSI. The Commission had undertaken efforts to bring about a relationship between NSOs and ETSI in the months leading up to ETSI’s inception (Temple 1991, 52). Prior to ETSI’s inception, the Commission communicated to the creator of ETSI, the European Conference of Postal and Telecommunications Administrations (CEPT), its preference that ETSI’s institutional architecture have “national structures underneath it, preferably the national standards bodies”. The European Commission’s preference was based on two key considerations: NSOs were ideally placed to hold national public enquiries for ETSI, and NSOs were equipped to transpose ETSI standards into the respective national standards. Temple notes that ETSI’s adherence to this specific preference of the European Commission’s led to NSOs being “wedged underneath ETSI without any direct agreement between the two principal parties” (53).

#### 3.2.2 Late 2010s

In July 2009, the European Commission published a White paper titled “Modernising ICT Standardization in the EU: the Way Forward” (hereinafter referred to as “the White paper”). The drivers behind the adoption of this White paper were the changes in the ICT standardization landscape, and specifically the emergence of specialized and mostly global fora and consortia as world-leading ICT standards development bodies. Technological changes also played an important role, as innovation shifted from the hardware to the software level. The European Commission sought to renew its ICT standardization policy to adapt to these developments and the needs of fast-moving markets, especially in services and high-technology products for fear that the EU no longer plays a key role in ICT standard setting, as the latter would mostly occur outside Europe (European Commission 2009, 2–3).

The EU White paper presented possible proposals for policy choices and specific measures to be included this future ICT policy. One proposal was the introduction of a list of attributes that ESO would need to respect in their standardisation processes to ensure that their standards developed in support of EU legislation and policies respect public policy objectives and needs. These principles were based on the WTO TBT criteria (Delimatsis 2019), which, the White paper noted, ESOs already observed them. Another proposal that was more controversial to ETSI was to enable the direct referencing to standards of specific fora and consortia (such as IETF, W3C and OASIS) in relevant EU legislation and policies, in order to fill specific standardization gaps. These organizations would need to fulfil the aforementioned attributes.

The European Commission would later establish an independent group named “Expert Panel for the Review of the European Standardisation System” (EXPRESS) “to review the entire ESS, its functioning, coherence, financing and legal framework and to deliver a report on the outlook for European standardisation in 2020” (European Commission and EXPRESS 2010, 8). ETSI perceived the White paper and the subsequent actions by the European Commission as indicative of its desire to “revise the whole policy environment for standardisation in Europe” (Weigel 2010, 9).

On 22 June 2010, ETSI communicated a Position Paper to Members of the EU Parliament (MEPs), which was soliciting inputs of stakeholders about the future of European standardization (ETSI 2010, 4). An important goal for ETSI in issuing this position was to make clear to these members how ETSI differed from CEN and CENELEC and that it was vital to consider these different set-ups in Europe. According to Weigel (2010), the ETSI Director-General (DG) at the time, there was a risk of ETSI being confounded with CEN and CENELEC. By issuing the position paper, ETSI sought to make the MEPs aware that the “three ESOs are not equal and their working methods are not based on the same principles” (Weigel 2010, 9).

In this position paper, ETSI emphasized that its unique set-up with direct-membership and a globalized approach was best suited to the needs of ICT standardisation. ETSI also explained how the ICT sector for which ETSI develops standards is not only intrinsically global and competitive but also of strategic importance to the EU in achieving its ambitions of a true pan-European service market. ETSI noted how ICT standards are intertwined with most if not all sectors on which the EU relies for its competitiveness. Cross-sector standardization needs increased co-operation between the ESOs, but this is easier said than done.

Regarding the challenge of dealing with standards from non-European countries, Weigel (2010) notes how giving external standards a European standardisation “blessing” was not the right way forward, and might raise legal issues related to the use of patents and the standards. Weigel emphasized the possibility of replicating the case of IEEE and Wi-Fi that had resulted in the publication of the wireless LAN standards as ETSI standards called HiperLAN as ways to overcome this challenge (Weigel 2010).

In a meeting with Mrs Kroes, the then European Digital Agenda Commissioner, Weigel also emphasized the global success of the GSM and 3GPP and its efforts to bring new work into ETSI. Weigel also noted the success of ETSI’s direct participation model in attracting the participation of SMEs and ETSI’s efforts to further encourage their participation, and the “need to overcome obstacles caused by the traditional segmentation of standards and by differences in approaches to standard making” (ETSI 2011, 10).

#### 3.2.3 Discussion

The findings of these two case studies illustrate how ETSI has taken a hard stance and resisted the desires of the European Commission for ETSI to make changes to its unique participation model and operational framework that would ensure greater involvement of national structures, preferably NSOs, in its standard setting work, also bringing ETSI in closer alignment with CEN and CENELEC’s set-up and practises. Under these conditions of contestation, ETSI has responded to the EU’s criticism and demands by enacting a strategy of communicating, awareness-raising and re-familiarizing the European Commission and other regulatory stakeholders about its unique set-up vis-à-vis that of CEN and CENELEC. ETSI has provided various arguments to justify and legitimize its choice to not change its set-up to the European Commission and its stakeholders.

The choice of the direct participation model has had profound implications for the scope of the role of NSOs within ETSI. Its lingering effects can be witnessed in the Amendment to Regulation 1025 (European Regulation 2022). As noted earlier, it appears that with this amendment to the Standardization Regulation, ETSI came full circle, leading to ETSI adopting new rules of procedure regarding its internal functioning when harmonized standards are mandated.^3^ Significantly, though, while the amendment gives a key role to NSO with regard to *voting*, the ETSI open governance model is maintained when it comes to the *promulgation* of harmonized standards. While our empirical research suggests that even within ETSI, high-level officials have become increasingly aware of the growing politicization of the standard-setting process, internal demands for fair treatment by non-European economic actors led to instances of contestation that eventually resulted in compromise where both sides could claim victory.

### 3.3 Case study for orchestration – ETSI’s IPR Policy

This case focuses on the interaction dynamics between the European Commission and ETSI when it established its first IPR Policy in 1993 and the subsequent revisions until 1995 when it received the European Commission’s favourable view on its then “interim IPR Policy”. This case sheds light on how ETSI was intentionally enrolled to serve the Commission’s preferences until ETSI adopted its 1994 Policy. More specifically, ETSI prioritized achieving compliance with the European Commission’s preferences on the Policy, embedded the European Commission’s language in the Policy, and swiftly undertook responsive measures prior to the European Commission’s action upon the receipt of a legal complaint against ETSI (European Commission 1995).

#### 3.3.1 Mission impossible? Navigating IPR issues among a heterogeneous ETSI membership

ETSI’s pursuance of an IPR Policy began in 1989, with the establishment of an Intellectual Property Rights Committee (IPRC) tasked with providing legal expertise in the formulation of the IPR Policy (Lee, Kim, and Kang 1999, 88). IPR issues had arisen during the standardisation of then Groupe Spécial Mobile (later renamed to Global System for Mobile Communication) GSM when a significant technical contributor, Motorola, refused to commit to the GSM Memorandum of Understanding (Iversen 1999). This case is illustrative of a problem that is well documented in the literature (Delimatsis, Kanevskaia, and Verghese 2021), of how conflicts over IPR and patents can complicate and slow down the standard setting process. In this case, such complications could potentially undermine the legitimacy of ETSI standards in the market.

This risk has increased over time with the proliferation of more complex technologies (Iversen 1999, 61). For ETSI, the direct participation model meant that its IPR Policy would necessarily have to strike a balance between protecting the interests of IP creators and contributors whilst ensuring that the terms of accessibility and licensing enable the implementers to access the technology in reasonable terms (ETSI 2001). This has meant that ETSI as an organization would have to adopt a relatively neutral approach whereby it offers a forum for discussions on licensing but does not take any resolute position in favour of licensors or licensees nor in favour of a single concept of FRAND licensing.

The European Commission being responsible for matters of competition law and antitrust enforcement within the EU, wished to avoid a situation wherein standards development at ETSI could be hijacked or abused by dominant interests to strengthen their market positions. In 1991, it adopted a statement in which it called on the European standards developers to develop “practical rules” that would bring clarity on the conditions under which IPR and patent are to be included in standards, and on rights associated with accessibility to such technologies “on free or on fair or reasonable terms” (European Commission 1990, 53). In 1992, it published a Communication setting out the principles which “should form the basis of any internal rules which standards bodies may wish to elaborate” (European Commission 1992). The Commission indicated that if a patent-holder chooses to make its SEPs available, “the terms for licenses must be fair, reasonable, and non-discriminatory”. The European Commission did not specify the meaning of “fair and reasonable”, however, indicating how specifying these terms was “not feasible or appropriate”, as these are “subjective factors determined by the circumstances surrounding the negotiations” (Stasik 2018, 77).

ETSI’s adoption of its first IPR Policy in 1993 gave rise to much controversy. This policy opted for an approach called “license by default” that went beyond the conditions on licensing proposed by the European Commission in its 1992 Communication. A controversial part of this policy also allowed IPR owners to inform ETSI of any specific IPRs that are to be exempted from being licensed on FRAND terms, provided that the refusal was notified to ETSI within 180 days of initiation of standards development work within ETSI's technical work program (Bekkers and Liotard 1999, 121). This provision was criticized on various accounts. For the European Commission, an important concern was that under this policy, an IPR holder would have a genuine possibility to withhold its IPRs; SSOs might not make sufficient information available to potential IPR holders and search processes for multinational companies could be too complex for searches to be conducted successfully and meaningfully (Iversen 1999).

Whilst an 88% majority within ETSI supported this approach, ETSI was experiencing considerable pressure from a minority group that disagreed with it. A North American “alliance” of companies was not willing “to obligate itself to license IPRs on what could potentially spread to a global scale while including their home market” (Iversen 1999). Certain disgruntled industry members threatened to quit ETSI (Iversen 1999). US and other national governments exerted pressure on stakeholders to withhold support for this policy. A decisive development was the legal complaint by the Computer and Business Equipment Manufacturers Association (CBEMA) submitted to the European Commission in June 1993, alleging that ETSI’s IPR Policy was in violation of EU competition law (Iversen 1999; Stasik 2018). According to Iversen (1999), it “suppressed implementation” of ETSI’s “license by default” approach. Before the European Commission could address this complaint, ETSI’s General Assembly abandoned the Undertaking and any reference thereto in the policy (Stasik 2018, 78). Our empirical research suggests that, at the time, active leadership exercised by the then DG and Deputy DG was instrumental in devising a compromise among the diverse, often conflicting interests but also the Commission’s expectations.

In November 1994, ETSI opted for an “interim IPR Policy”, under which willing members could agree to license their patented technologies embedded in ETSI deliverables on FRAND terms. This amended version of ETSI’s 1993 IPR Policy, borrowed the European Commission’s language regarding key concepts, abandoned the contentious component, and retained specific obligations that ETSI perceived as being “not at all onerous” (ETSI 2001). The policy was adopted as part of the ETSI Rules of Procedure, thus binding all ETSI members to the rights and obligations in the Policy (ETSI 2001).

Since the 1993 undertaking was abandoned, and the availability of licenses under FRAND governed by Section 6.1, ETSI has resisted all attempts to further define ‘fair, reasonable, and non-discriminatory’ beyond the plain, literal meaning of those words as they were handed down from the European Commission and placed into the ETSI IPR Policy. (Stasik 2018, 79)

Although since its adoption in 1994, ETSI’s IPR Policy has undergone revisions and continues to attract much debate both within and outside courts and among industry stakeholders and interested parties, the “fundamental features and language” of the most recent version of ETSI’s IPR Policy remains “identical” to that of the “interim” version developed nearly thirty years ago (Pocknell and Djavaherian 2023, 992). ETSI’s IPR Policy is noted for its brevity. Although, as our empirical research suggests, pressure was exercised on ETSI to take steps on further elaborating its IPR Policy, ETSI has steadfastly refused to entertain calls to further elaborate on the FRAND terms (Brooks and Geradin 2010, quoted in Stasik 2018, 79), often relying on the justification that it does not wish to become party to what ETSI deems as being essentially commercial matters. Originally, when most patentholders in the field were European, the ETSI deferential approach, one could argue, made sense from a strategic viewpoint, as it would favour European innovators. As things evolved and innovative technologies were created increasingly outside Europe, the FRAND question became a global matter. The member-driven nature of ETSI, however, but also the complexity of the ICT landscape, which often requires cross-licensing to function, would again speak in favour of a neutral stance on the side of ETSI, according to our empirical findings.

#### 3.3.2 Discussion

The case study illustrates how ETSI intentionally acquiesced to the European Commission’s desires or preferences. ETSI abandoning the contentious components in its IPR Policy amidst pressures, and before the European Commission could formally address the legal complaint, can be viewed as an orchestration strategy. In doing so, ETSI diffused criticism and could reach a consensus within its membership.

This change enhanced ETSI’s legitimacy in the eyes of members, especially the ones that held dissenting and critical views on the prior versions of the IPR Policy. The rationale behind the choice(s) made by ETSI *in enacting this change* are based on *legitimacy considerations and in view of legal compliance*. The assumption is that the consequences of this interaction enhanced public action, but also affected favourably ETSI’s resilience and external legitimacy. Internally, it also strengthened ETSI as a neutral forum for negotiations and discussions among competing interests.

ETSI’s adoption of the IPR Policy of 1994 was the culmination of close to five years of intensive discussions (Pocknell and Djavaherian 2023, 114) of around “50 legal experts representing (amongst others) technology owners and manufacturers from Europe and overseas” (Rosenbrock 2017). The European Commission was closely involved in the development of ETSI’s IPR Policy to ensure, amongst other thing, compliance with EU law and especially competition law (Bekkers 2001). According to Bekkers (2001, 249), “during the policy development stage, the Commission regularly intervened with indications of what it would and would not find acceptable”.

This case, taking place in an SDOs formative years (within the first six years of ETSI), is also illustrative of how a regulator’s hands-on yet limited regulatory intervention (Stasik 2018, 73) in discussing topics and concepts pertinent to ETSI’s internal procedural and policy matters (Iversen 1999) made it possible for ETSI to take a relatively hands-off stance, once it managed to stem the controversy on what may essentially be an intractable topic of the IPR Policy.

### 3.4 Case study for collaboration – the GSM “SIM Lock” case

#### 3.4.1 A story of harmonious collaboration

Primarily purposed as a “theft deterrent” mechanism (Hillebrand 2002), the Subscriber Identity Module Lock (“SIM Lock”) feature could be used by the mobile communications service providers to restrict the customers’ freedom to link their own handset to a competitor’s network. Regulatory concerns arose regarding the latter functionality, particularly owing to the potential for negatively impacting consumer choice, and in serving as a barrier to entry of new competitors (Analysys Ltd., and Squire, Sanders & Dempsey L.L.P. 1998, 228). The European Commission was also concerned regarding European customers having to incur significant expenses and inconvenience in availing the services of a competitor; as well as regarding the feature's potential to intensify the structuring of the European mobile phone market along national lines, and thus jeopardizing the climate of competition (European Commission 1996).

In May 1996, the European Commission's Directorate-General for Competition [DG IV] sent a “warning letter” regarding the feature’s anti-competitive effect to the network operators and manufacturers (Gregson 1996). In July 1996, the Commission concluded its investigation and allowed manufacturers to use the “SIM Lock” feature, as long as the customer was made aware of the feature at the time of purchase of the device, and that design provisions allowed for unlocking by the customer or on demand (Hillebrand 2002, 83).

The European Commission shared a copy of the letter with ETSI which was, at the time, proposing to include the “SIM Lock” feature into the GSM Technical Specification. The European Commission's investigations showed that the proposal on including the feature lacked enthusiasm among a majority of the telecom operators. The European Commission indicated that ETSI incorporates the “SIM Lock” feature into the technical specification after having carefully considered the feature from a consumer-centric perspective (Hillebrand 2002).

ETSI’s General Assembly constituted an “Ad Hoc Group for Consultation with the European Commission” comprising of the then ETSI Deputy DG, and ETSI representatives from the technical committee concerned, the ETSI Secretariat, and European and global mobile services industry/ trade associations. The Group and the European Commission held a meeting on 25 July 1996, where the European Commission “asked thoroughly” on the benefits, and applications of the feature, and “intensively” (Hillebrand 2002) on the scope for its substitutability with another technology (GSMA 2014). ETSI in its responses stated that the proposed clause for the feature complied with the European Commission's preference, noted that the substitutable initiative did not enjoy optimal levels of voluntary contributions, and offered the following rationale for the inclusion of the clause on the “SIM Lock” feature:

The group stressed, that ETSI as an organisation based in Europe produces the GSM standard for the world market and that the possibility must exist to produce standards to the needs of all regions, whether their use is fully compatible with European law or not. (Hillebrand 2002)

The European Commission noted the above-mentioned rationale and, while agreeing with ETSI, emphasized its obligation for scrutinizing compliance with EU law, particularly EU competition rules. Subsequently, ETSI in its letter dated 30 July 1996, sought the European Commission's confirmation that ETSI could continue its subsequent work on SIM Lock to create “coherence” between “the information received by the manufacturers and the corresponding standardisation activities” (Hillebrand 2002).

However, the relevance of this episode for our purposes lies in the further developments that would ensue. Although the European Commission and ETSI agreed on initiating earlier consultations for future cases of concerns (Hillebrand 2002), the European Commission conveyed the concern that its “attention had first and foremost been raised by the inclusion in the deliverables concerned of terms which were obviously anti-competitive” (ETSI 2000). The European Commission in its letter dated 7 August 1996 “invited” ETSI to assess the impact of its decisions on competition “at an early stage before their final implementation” (ETSI 2000).

In August 1996, ETSI followed the European Commission's recommendation “to proceed to an assessment of competitive impact of ETSI standards” by undertaking a legal review of pertinent deliverables (approximately 200 documents) with the objective to “limit the risk of future interventions of the Commission DGIV (Competition law division), on matters related to the deliverables” (Hillebrand 2002). ETSI combed through its documents to isolate references and agreements that were potentially (1) anti-competitive (Article 85 of the EC Treaty, now 101 TFEU); (2) leading to abuse of dominant position and; (3) in breach of obligations flowing from Directive 83/189 as applicable to ESOs, especially pertaining to inclusion of commercial schemes and non-admissibility of sole reliance on technical arguments. ETSI subsequently worked with its technical experts to assess the necessity of retaining the flagged portions, and so, to make the appropriate amendments. Although ETSI identified a few provisions, all save for one were eventually determined to be legal, and this one provision was deleted (Hillebrand 2002).

Furthermore, ETSI acknowledged the inherent vulnerability of its arena for use by actual or potential competitors to meet and decide on anti-competitive action and noted that “competition cases could have been avoided if people had been educated” (ETSI 2000). ETSI would subsequently prepare to undertake precautionary efforts by establishing a “Competition Law Compliance Programme” for its staff members and experts from 1997 onwards (European Commission, Schepel, and Falke 2000, 134). A crucial component of this Programme is the “Guide to European Competition Law” which described in an accessible manner the implications of anti-competitive agreements, abuses of dominant position, consequences of infringements of Articles 81 and 82, and on how to avoid the adoption of anti-competitive agreements in ETSI (ETSI 2000).

ETSI personnel did not consider the SIM Lock case to be a “crisis” event, nor was it accorded as a moment as having affected ETSI’s legitimacy. However, we refer to this instance given its highly illustrative representation of collaborative interactions between ETSI and the European Commission on such an important matter for the ICT landscape.

#### 3.4.2 Discussion

ETSI's engagement with the European Commission during the SIM Lock case is characterized as a collaboration strategy. Firstly, ETSI swiftly reviewed internal documentation for any clauses that could be interpreted as being potentially facilitative of anti-competitive practices. Secondly, ETSI utilized the interactions with the European Commission as an avenue to inform the regulator of ETSI's difficult balancing act of managing conflicting goals while developing standards. Thirdly, ETSI also took steps to ensure that its deliverables did not cause a reduction in the choice available to an average European user of telecommunication services. ETSI's swiftness in reviewing its documentation conveys responsiveness to address the European Commission's concerns. ETSI's transparency in its motivations for inclusion of SIM Lock provisions – that its technical specifications can accommodate SIM Lock provisions outside of the EU when a mobile operator chooses to do so lawfully – were also accompanied by its commitment to EU Single Market preferences on ensuring that there are no constrains on the choice available to the European user.

Other than effectively collaborating with the European Commission to achieve a common goal, ETSI’s actions can be viewed as an effective means to enhance its resilience via ETSI’s acknowledgment that whilst it was indirectly accountable to the European Commission, it also had a mandate to serve the standardisation needs of its members; in having communicated so, ETSI accomplished providing both the members and the European Commission with clarity. The series of responsive steps, infusing clarity with regulators, and procedural checks enhanced ETSI’s legitimacy in the eyes of its members (most notably of the mobile operators operating in emerging economies) as well as from European Commission.

The rationale behind the choices made by ETSI was to strike a balance between the commercial interests of its members (mobile operators), and the regulatory preferences of the European Commission. The assumption is that the consequences of this interaction resulted in enhanced public action, namely in the European Commission’s role in proactively communicating its concerns on the potential misuse of the SIM Lock provisions within EU geography, and in ETSI’s actions.

## 4 Conclusion

This article offered a glance into the resilience dynamics of standards development organizations, using ETSI as an example. It argued that ETSI used various strategies to sustain its organizational resilience over the years. However, in its interaction with its de facto controller, the European Commission, in particular, ETSI had recourse to three key resilience strategies: one where ETSI took a hard stance resisting to the Commission’s desires (contestation), one where ETSI intentionally succumbed to the Commission’s desires (orchestration), and one where ETSI opted for a reconciliatory approach to achieve commonly set objectives serving mutual interest (collaboration). Interestingly, such strategies were not only resilience-enhancing but also legitimacy-conducive, reinforcing further ETSI’s position as the preferred ICT standard-setter at the EU level and a reliable global SDO that manages the balance between the expectations of EU institutions and the ambition of its global membership.

We argued that by increasing its resilience through implementing these three strategies, ETSI was also reinforcing its legitimacy as it was reacting to internal legitimacy demands. Thus, the article contributes to these two different strings of academic literature relating to organizational resilience, on the one hand, and institutional legitimacy, on the other, by combining them in a manner that, to the best of our knowledge, had not been done before in the relevant literature. This article further aspires to instigate more empirical research to further unveil critical moments of interaction between ETSI (but also the other ESOs) and the European Commission. As the latter hardens its stance vis-à-vis private regulators and as we move to more strategic and geopolitical considerations informing such interactions, empirical accounts can allow drawing lessons regarding successful instances but also instances which in retrospect could have led to better, more efficient outcomes.

Meticulous research is all the more warranted as one should avoid throwing out the baby with the bathwater: ETSI, with all its birth defects and imperfections, has evolved into the crown jewel of the EU standardization system, drawing from within the EU when required and broadening its partnerships outside the EU when applicable. The latter, while risky geopolitically, also undoubtedly increased ETSI’s influence beyond the EU’s borders. As international standardization takes a techno-nationalistic turn and a new dynamic unfolds to global cooperation and competition for setting the global rules for the next-generation technologies, from AI to 5G (Kim, Doyoung, and Lee 2023), how the European Commission interacts with ETSI will have major repercussions for EU’s relative competitiveness globally.

## Notes

1 Discussing the variants of co-regulation is outside the scope of this article.

2 ERC project REVEAL (Grant Agreement No 725798-REVEAL), more information at: https://www.tilburguniversity.edu/research/institutes-and-research-groups/tilec/research/projects-networks/reveal.

3 See also Volpato and Eliantonio (2024) in this Special Issue.

## References

[CIT0001] Abbott, Kenneth W., Philipp Genschel, Duncan Snidal, and Bernhard Zangl, eds. 2015. *International Organisations as Orchestrators*. Cambridge: Cambridge University Press. 10.1017/CBO9781139979696.

[CIT0002] Abbott, Kenneth W., Philipp Genschel, Duncan Snidal, and Bernhard Zangl. 2016. “Two Logics of Indirect Governance: Delegation and Orchestration.” *British Journal of Political Science* 46 (4): 719–729. 10.1017/S0007123414000593.

[CIT0003] Alexandridis, Stylianos. 2019. “ETSI: A Case Study in Standard Setting and Capture.” PhD diss., Queen Mary, University of London. https://qmro.qmul.ac.uk/xmlui/handle/123456789/79101.

[CIT0004] Analysys Ltd., and Squire, Sanders & Dempsey L.L.P. 1998. Adapting the EU Regulatory Framework to the Developing Multimedia Environment. A Study for the European Commission: Annex I: Comparative Overview of Current Regulatory Environment in Telecommunications and Broadcasting Sectors. (Brussels-Luxembourg: European Commission, Directorate General XIII). http://aei.pitt.edu/id/eprint/43251.

[CIT0005] Anstar Oy vs Turvallisuus-ja kemikaalivirasto (Tukes). 2017. Case C-630/16 Judgment of 14 December 2017. https://eur-lex.europa.eu/legal-content/EN/TXT/PDF/?uri=CELEX:62016CJ0630&from=EN.

[CIT0006] Bartley, Tim. 2021. “Power and the Practice of Transnational Private Regulation.” *New Political Economy* 27 (2): 188–202. 10.1080/13563467.2021.1881471.

[CIT0007] Bekkers, Rudi. 2001. *Mobile Telecommunications Standards: GSM, UMTS, TETRA, and ERMES*. London: Artech House.

[CIT0008] Bekkers, Rudi, and Isabelle Liotard. 1999. “European Standards for Mobile Communications: The Tense Relationship Between Standards and Intellectual Property Rights.” *European Intellectual Property Review* 21 (3): 110–126. https://hal.science/hal-00351198.

[CIT0009] Biddle, Bradford. 2017. “No Standards for Standards: Understanding the ICT Standards-Development Ecosystem.” In *The Cambridge Handbook of Technical Standardization Law – Competition, Antitrust and Patents*, edited by Jorge Contreras, 17–28. Cambridge: Cambridge University Press. 10.1017/9781316416723.004.

[CIT0010] Black, Julia. 2008. “Constructing and Contesting Legitimacy and Accountability in Polycentric Regulatory Regimes.” *Regulation & Governance* 2 (2): 137–164. 10.1111/j.1748-5991.2008.00034.x.

[CIT0011] Cantero Gamito, Marta. 2018. “Europeanization Through Standardization: ICT and Telecommunications.” *Yearbook of European Law* 37 (1): 395–423. 10.1093/yel/yey018.

[CIT0012] Cashore, Benjamin, Jette Steen Knudsen, Jeremy Moon, and Hamish van der Ven. 2021. “Private Authority and Public Policy Interactions in Global Context: Governance Spheres for Problem Solving.” *Regulation & Governance* 15 (4): 1166–1182. 10.1111/rego.12395.

[CIT0013] David, Paul A., and Mark Shurmer. 1996. “Formal Standards-Setting for Global Telecommunications and Information Services. Towards an Institutional Regime Transformation?” *Telecommunications Policy* 20 (10): 789–815. 10.1016/S0308-5961(96)00060-2.

[CIT0014] Delimatsis, Panagiotis. 2015. “‘Relevant International Standards’ and ‘Recognised Standardisation Bodies’ under the TBT Agreement.” In *The Law, Economics and Politics of International Standardisation*, edited by Panagiotis Delimatsis, 104–136. Cambridge: Cambridge University Press. 10.1017/cbo9781316423240.006.

[CIT0015] Delimatsis, Panagiotis. 2018. “Global Standard-Setting 2.0: How the WTO Spotlights ISO and Impacts the Transnational Standard-Setting Process.” *Duke Journal of Comparative & International Law* 28 (2): 273–326. https://scholarship.law.duke.edu/djcil/vol28/iss2/3.

[CIT0016] Delimatsis, Panagiotis. 2019. “International Trade Law and Technical Standardization.” In *The Cambridge Handbook of Technical Standardization Law: Further Intersections of Public and Private Law*, edited by Jorge L. Contreras, 7–27. Cambridge: Cambridge University Press. 10.1017/9781316416785.002.

[CIT0017] Delimatsis, Panagiotis. 2023a. “The Resilience of Private Authority in Times of Crisis.” In *The Evolution of Transnational Rule-Makers through Crises*, edited by Panagiotis Delimatsis, Stephanie Bijlmakers, and M. Konrad Borowicz, 21–46. Cambridge: Cambridge University Press. 10.1017/9781009329408.003.

[CIT0018] Delimatsis, Panagiotis. 2023b. “Transnational Economic Activism and Private Regulatory Power.” *Journal of International Economic Law* 26 (3): 559–576. 10.1093/jiel/jgad028.

[CIT0019] Delimatsis, Panagiotis, Stephanie Bijlmakers, and M. Konrad Borowicz. 2023. “Introduction: How Private Rule-Makers Evolve through Crises.” In *The Evolution of Transnational Rule-Makers through Crises*, edited by Panagiotis Delimatsis, Stephanie Bijlmakers, and M. Konrad Borowicz, 1–18. Cambridge: Cambridge University Press. 10.1017/9781009329408.001.

[CIT0020] Delimatsis, Panagiotis, O. Kanevskaia, and Z. G. Verghese. 2021. “Strategic Behavior in Standards Development Organizations in Times of Crisis.” *Texas Intellectual Property Law Journal* 29 (2): 127–185. https://tiplj.org/wp-content/uploads/Volumes/v29/Delimatsis_final.pdf.

[CIT0021] Eliantonio, Mariolina, and Annalisa Volpato. 2022. “The European System of Harmonised Standards. Legal Opinion for ECOS.” *SSRN Electronic Journal* 1–40. 10.2139/ssrn.4055292.

[CIT0022] ETSI (European Telecommunications Standards Institute). 2000. *A Guide to European Competition Law.* https://web.archive.org/web/20000815210447/http://www.etsi.org/handbook/comp_law.htm.

[CIT0023] ETSI (European Telecommunications Standards Institute). 2001. *The Technical Body Chairman's Guide.* https://web.archive.org/web/20011218130115/http://www.etsi.org/legal/ipr_guide.htm#Historical%20background.

[CIT0024] ETSI (European Telecommunications Standards Institute). 2010. “European Standardization: A Way out of the Economic Crisis.” *The Standard ETSI Newsletter*. September 2010. https://www.etsi.org/images/files/ETSInewsletter/ETSI%20eNewsletter_September_2010.pdf.

[CIT0025] ETSI (European Telecommunications Standards Institute). 2011. “Supporting the Digital Agenda.” *The Standard ETSI Newsletter* February 2011. https://www.etsi.org/images/files/ETSInewsletter/ETSINewsletter_FEB2011.pdf.

[CIT0026] ETSI (European Telecommunications Standards Institute). 2023. *ETSI Directives Version 47 30 June* 2023. Sophia Antipolis, France: European Telecommunications Standards Institute. https://web.archive.org/web/20231001021017/https://portal.etsi.org/Resources/ETSI-Directives.

[CIT0027] European Commission. 1985. *Completing the Internal Market – White Paper from the Commission to the European Council COM(85) 310 final 14 June 1985*. Luxembourg: Office for Official Publications of the European Communities. https://op.europa.eu/s/zgCb.

[CIT0028] European Commission. 1990. *Commission Green Paper on the Development of European Standardization: Action for Faster Technological Integration in Europe* COM (90) 456 final – 08 October 1990. OJ C C/20. https://eur-lex.europa.eu/legal-content/EN/TXT/?uri=CELEX:51990DC0456.

[CIT0029] European Commission. 1992. *Communication from the Commission - Intellectual Property Rights and Standardization* COM(92) 445 final – 27 October 1992. https://op.europa.eu/s/zgNd.

[CIT0030] European Commission. 1995. *Notice Pursuant to Article 19 (3) of Council Regulation No 17 concerning Case No IV/35.006 - ETSI Interim IPR Policy –* 28 March 1995. Official Journal C/76 5–7. https://eur-lex.europa.eu/legal-content/EN/TXT/?uri=CELEX:C1995/076/05.

[CIT0031] European Commission. 1996. The Commission Takes Action to Prevent Anti-Competitive Practices in the Mobile Phones Sector IP/96/791. https://ec.europa.eu/commission/presscorner/detail/en/IP_96_791.

[CIT0032] European Commission. 2003. “*General Guidelines for the Cooperation between CEN, Cenelec and ETSI and the European Commission and the European Free Trade Association* — 28 March 2003.” *Official Journal of the European Union C* 91:7–11. https://eur-lex.europa.eu/legal-content/EN/TXT/?uri=CELEX:52003XC0416(03).

[CIT0033] European Commission. 2009. *White Paper: Modernising ICT Standardization in the EU - The Way Forward* COM(2009) 324 final – 03 July 2009. https://op.europa.eu/s/zgCH.

[CIT0034] European Commission. 2011. *Communication from the Commission to the European Parliament, the Council and the European Economic and Social Committee: A Strategic Vision for European Standards: Moving Forward to Enhance and Accelerate the Sustainable Growth of the European Economy by 2020* COM(2011) 311 final 01 June 2011. https://op.europa.eu/s/zgCc.

[CIT0035] European Commission. 2015. *Vademecum on European Standardisation in Support of Union Legislation and Policies: Part II Preparation and Adoption of the Commission’s Standardisation Requests to the European Standardisation Organisations* SWD(2015) 205 final 27 October 2015. https://ec.europa.eu/docsroom/documents/13508/attachments/1/translations/en/renditions/pdf.

[CIT0036] European Commission. 2020. *Communication from the Commission to the European Parliament, the European Council, the Council, the European Economic and Social Committee and the Committee of the Regions: A New Industrial Strategy for Europe* COM(2020) 102 final 10 March 2020. https://eur-lex.europa.eu/legal-content/EN/TXT/?uri=CELEX:52020DC0102.

[CIT0037] European Commission. 2021. *Study on the Implementation of the Regulation (EU) No. 1025/2012 (Article 24) – Final Report* — 15 October 2020. Luxembourg: Publications Office of the European Union. 10.2873/504681.

[CIT0038] European Commission. 2022a. *Communication from the Commission to the European Parliament, the Council, the European Economic and Social Committee and the Committee of the Regions: An EU Strategy on Standardisation Setting Global Standards in Support of a Resilient, Green and Digital EU Single Market* COM(2022) 31 final 02 February 2022. https://op.europa.eu/s/zgCE.

[CIT0039] European Commission. 2022b. “Feedback from: Fraunhofer IIS Amendment to the Regulation (EU) No 1025/2012 European Standardisation.” European Commission. https://ec.europa.eu/info/law/better-regulation/have-your-say/initiatives/13376-Amendment-to-the-Regulation-EU-No-1025-2012-European-standardisation/F3053752_en.

[CIT0040] European Commission and Expert Panel for the Review of the European Standardization System (EXPRESS). 2010. *Report of the Expert Panel for the Review of the European Standardization System, Standardization for a Competitive and Innovative Europe: A Vision for* 2020 EXP 384 *final.* https://www.europarl.europa.eu/document/activities/cont/201006/20100611ATT75994/20100611ATT75994EN.pdf.

[CIT0041] European Commission, Harm Schepel, and Josef Falke. 2000. *Legal aspects of standardisation in the Member States of the EC and EFTA. Volume 1, Comparative report*. Vol. 1. Luxembourg: Office for Official Publications of the European Communities. https://op.europa.eu/s/zgCG.

[CIT0042] European Council. 1985. *Council Resolution of 7 May 1985 on a New Approach to Technical Harmonization and Standards.* Official Journal C 136 1–9. Luxembourg: Office for Official Publications of the European Communities. https://eur-lex.europa.eu/legal-content/EN/TXT/?uri=CELEX:31985Y0604(01)).

[CIT0043] European Regulation. 2012. *(EU) No 1025/2012 of the European Parliament and of the Council of 25 October 2012 on European Standardisation* OJ L 316/12 14 November 2012. Official Journal of the European Union. http://data.europa.eu/eli/reg/2012/1025/oj.

[CIT0044] European Regulation 2022. *(EU) No 2022/2480 of the European Parliament and of the Council of 14 December 2022 amending Regulation (EU) No 1025/*2012 OJ L 323/1 19 December 2022. Official Journal of the European Union. https://eur-lex.europa.eu/eli/reg/2022/2480/oj.

[CIT0045] Falke, Josef. 1997. “Achievements and Unresolved Problems of European Standardization: The Ingenuity of Practice and the Queries of Lawyers.” In *Integrating Scientific Expertise into Regulatory Decision-Making: National Traditions and European Innovations*, edited by Christian Joerges, Karl-Heinz Ladeur, and Ellen Vos, 187–224. Baden-Baden, Germany: Nomos.

[CIT0046] Farrell, Joseph, and Garth Saloner. 1988. “Coordination through Committees and Markets.” *The RAND Journal of Economics* 19 (2): 235–252. https://www.jstor.org/stable/2555702.

[CIT0047] Fra.bo SpA v Deutsche Vereinigung des Gas- und Wasserfaches eV (DVGW) — Technisch- Wissenschaftlicher Verein. 2012. C-171/11 ECLI:EU:C:2012:453. https://curia.europa.eu/juris/liste.jsf?num=C-171/11&language=EN.

[CIT0048] Freeman, Jody. 2000. “Private Parties, Public Functions and the New Administrative Law.” *Administrative Law Review* 52 (3): 813–858. http://www.jstor.org/stable/40711903.

[CIT0049] Gandal, Neil, and Pierre Régibeau. 2015. “Standard-Setting Organisations: Current Policy Issues and Empirical Evidence”. In *The Law, Economics and Politics of International Standardisation*, edited by Panagiotis Delimatsis, 394–433. Cambridge: Cambridge University Press. 10.1017/CBO9781316423240.017.

[CIT0050] Gregson, Reily. 1996. “European Commission warns Carriers about SIM Card Lock Use.” *RCR*, June 24, 1996. https://www.rcrwireless.com/19960624/archived-articles/european-commission-warns-car-riers-about-sim-card-lock-use.

[CIT0051] GSMA (GSM Association). 2014. IMEI Database. https://www.gsma.com/latinamerica/wp-content/uploads/2014/11/IMEI-Database-Overview.pdf.

[CIT0052] Hale, Thomas, and Charles Roger. 2014. “Orchestration and Transnational Climate Governance.” *The Review of International Organizations* 9 (1): 59–82. 10.1007/s11558-013-9174-0.

[CIT0053] Henriksen, L. F., and Stefano Ponte. 2018. “Public Orchestration, Social Networks, and Transnational Environmental Governance: Lessons from the Aviation Industry.” *Regulation & Governance* 12 (1): 23–45. 10.1111/rego.12151.

[CIT0054] Hillebrand, Friedhelm. 2002. “Evolving the Services and System Features to Generation 2.5 by the GSM Phase 21 Programme (1993–2000): The GSM Work in ETSI SMG from May 1996 to July 2000.” In *GSM and UMTS: The Creation of Global Mobile Communication*, edited by Friedhelm Hillebrand, 80–98. Chichester: John Wiley & Sons.

[CIT0055] Iversen, Erik J. 1999. “Standardization and Intellectual Property Rights: ETSI's Controversial Search for New IPR-Procedures.” In *Proceedings of 1st International IEEE Conference on Standardisation and Innovation in Information Technology (SIIT ‘99), Aachen, Germany, September 15-17, 1999*, edited by K. Jakobs, and R. Williams, 55–64. Piscataway, NJ: IEEE. https://hdl.handle.net/102.100.100/493487.

[CIT0056] James Elliott Construction Limited v Irish Asphalt Limited. 2016. C-613/14 ECLI:EU:C:2016:821. https://curia.europa.eu/juris/liste.jsf?language=en&num=C-613/14.

[CIT0057] Kanevskaia, Olia. 2023. *The Law and Practice of Global ICT Standardization*. Cambridge: Cambridge University Press. 10.1017/9781009300551.

[CIT0058] Kim, Mi-jin, Eom Doyoung, and Heejin Lee. 2023. “The Geopolitics of Next Generation Mobile Communication Standardization: The Case of Open RAN.” *Telecommunications Policy* 47 (10): 102625. 10.1016/j.telpol.2023.102625.

[CIT0059] Knudsen, J. S., and J. Moon. 2017. *Visible Hands: Government Regulation and International Business Responsibility*. Cambridge: Cambridge University Press. 10.1017/9781316224908.

[CIT0060] Larouche, Pierre, and Florian Schuett. 2019. “Repeated Interaction in Standard Setting.” *Journal of Economics & Management Strategy* 28 (3): 488–509. 10.1111/jems.12287.

[CIT0061] Lee, S. M., Y. T. Kim, and B. M. Kang. 1999. “Analysis of the Change of ETSI IPR Policies.” *Electronics and Telecommunications Trends* 14 (3): 86–94. 10.22648/ETRI.1999.J.140308.

[CIT0062] Lerner, Josh, and Jean Tirole. 2015. “Standard-Essential Patents.” *Journal of Political Economy* 123 (3): 547–586. 10.1086/680995.

[CIT0063] Mattli W., and J. Seddon. 2015. “The Power of the Penholder: The Missing Politics in Global Regulatory Governance Analysis.” In *The Law, Economics and Politics of International Standardisation* edited by Panagiotis Delimatsis, 169–198. Cambridge: Cambridge University Press. 10.1017/CBO9781316423240.009.

[CIT0064] Pocknell, Robert, and David Djavaherian. 2023. “The History of the ETSI IPR Policy: Using the Historical Record to Inform Application of the ETSI FRAND Obligation.” *Rutgers University Law Review* 75 (3): 977–1010. https://heinonline.org/HOL/P?h=hein.journals/rutlr75&i=1015.

[CIT0065] Rosenbrock, Karl Heinz. 2017. *Why the ETSI IPR Policy Requires Licensing to All.* http://www.fair-standards.org/wp-content/uploads/2017/08/Why-the-ETSI-IPR-Policy-Requires-Licensing-to-All_Karl-Heinz-Rosenbrock_2017.pdf.

[CIT0066] Roux-Dufort, C., and C. Lalonde. 2013. “Editorial: Exploring the Theoretical Foundations of Crisis Management.” *Journal of Contingencies and Crisis Management* 21 (1): 1–3. 10.1111/1468-5973.12014.

[CIT0067] Rysman, Marc, and Timothy Simcoe. 2008. “Patents and the Performance of Voluntary Standard-Setting Organizations.” *Management Science* 54 (11): 1920–1934. 10.1287/mnsc.1080.0919.

[CIT0068] Schepel, Harm. 2005. *The Constitution of Private Governance: Product Standards in the Regulation of Integrating Markets*. Oxford: Hart Publishing.

[CIT0069] Schepel, Harm. 2013. “The New Approach to the New Approach: The Juridification of Harmonized Standards in EU Law.” *Maastricht Journal of European and Comparative Law* 20 (4): 521–533. 10.1177/1023263X1302000404.

[CIT0070] Schepel, Harm. 2015. “Between Standards and Regulation.” In *The Law, Economics and Politics of International Standardisation*, edited by Panagiotis Delimatsis, 199–214. Cambridge: Cambridge University Press. 10.1017/CBO9781316423240.010.

[CIT0071] Senden, Linda. 2017. “The Constitutional Fit of European Standardization Put to the Test.” *Legal Issues of Economic Integration* 44 (Issue 4): 337–352. 10.54648/LEIE2017018.

[CIT0072] Sheffner, Daniel J. 2019. “Integrating Technical Standards into Federal Regulations: Incorporation by Reference.” In *The Cambridge Handbook of Technical Standardization Law: Further Intersections of Public and Private Law*, edited by Jorge L. Contreras, 108–123. Cambridge: Cambridge University Press. 10.1017/9781316416785.007.

[CIT0073] Stanojević, Antonia. 2024. “ETSI as a Case Study of Organizational Resilience in Standard Setting: Strategies That Ensure Thriving Despite Organizational Challenges.” *Innovation: The European Journal of Social Science Research* 1–15. 10.1080/13511610.2024.2369180. PMC11841373 39981503

[CIT0074] Stasik, Eric L. 2018. “The Role of the European Commission in the Development of the ETSI IPR Policy and the Nature of FRAND in Standardization.” In *Multi-dimensional Approaches Towards New Technology: Insights on Innovation, Patents and Competition*, edited by Ashish Bharadwaj, Vishwas H. Devaiah, and Indranath Gupta, 73–83. Singapore: Springer. 10.1007/978-981-13-1232-8_4.

[CIT0075] Stichting Rookpreventie Jeugd and Others v Staatssecretaris van Volksgezondheid, Welzijn en Sport, C-160/20 ECLI:EU:C:2022:101. 2022. https://e-justice.europa.eu/ecli/ECLI:EU:C:2022:101.

[CIT0076] Temple, Stephen. 1991. *European Telecommunications Standards Institute: A Revolution in European Telecommunications Standards Making*. Hull, UK: Kingston Public Relations.

[CIT0077] Tosun, Jale, Sebastian Koos, and Jennifer Shore. 2016. “Co-Governing Common Goods: Interaction Patterns of Private and Public Actors.” *Policy and Society* 35 (1): 1–12. 10.1016/j.polsoc.2016.01.002.

[CIT0078] Van Gestel, Rob, and H-W. Micklitz. 2013. “European Integration Through Standardization: How Judicial Review is Breaking Down the Club House of Private Standardization Bodies.” *Common Market Law Review* 50 (1): 145–181. 10.54648/cola2013007.

[CIT0079] Volpato, Annalisa, and Mariolina Eliantonio. 2024. “The Participation of Civil Society in ETSI from the Perspective of Throughput Legitimacy.” *The European Journal of Social Science Research* 1–17. 10.1080/13511610.2024.2321852.

[CIT0080] Weigel, W. 2010. “ETSI: Exceptionalism in Future European Standards”. *InterComms* October 2010 15: 9-10. https://www.intercomms.net/issue-15/pdfs/articles/etsi.pdf.

[CIT0081] Wiener, Antje. 2014. *A Theory of Contestation*. Heidelberg: Springer Berlin. 10.1007/978-3-642-55235-9.

[CIT0082] Wolbers, Jeroen, Sanneke Kuipers, and Arjen Boin. 2021. “A Systematic Review of 20 Years of Crisis and Disaster Research: Trends and Progress.” *Risk, Hazards & Crisis in Public Policy* 12 (4): 374–392. 10.1002/rhc3.12244.

[CIT0083] WTO Committee on Technical Barriers to Trade (TBT Committee). 2000. *Second Triennial Review of the Operation and Implementation of the Agreement on Technical Barriers to Trade: Annex 4 Decision of the Committee on Principles for the Development of International Standards, Guides and Recommendations with Relation to Articles 2, 5 and Annex 3 of the Agreement*. G/TBT/9. Accessed April 22, 2024. https://docs.wto.org/dol2fe/Pages/FE_Search/FE_S_S009-DP.aspx?language=E&CatalogueIdList=231,4879&CurrentCatalogueIdIndex=1.

[CIT0084] Zürn, Michael. 2018. *A Theory of Global Governance: Authority, Legitimacy, and Contestation*. Oxford: Oxford University Press. 10.1093/oso/9780198819974.001.0001.

